# The effect of cognitive-behavioral counseling on anxiety in the mothers of infants in the NICU:  A randomized controlled trial

**DOI:** 10.12688/f1000research.12539.1

**Published:** 2017-09-12

**Authors:** Massumeh Koochaki, Zohreh Mahmoodi, Sara Esmaelzadeh – Saeieh, Kourosh Kabir, Maryam Tehranizadeh, Mahrokh Dolatian

**Affiliations:** 1 Alborz University of Medical Sciences, Karaj, Iran; 2 Payame Noor University, Karaj, Iran; 3 Shahid Beheshti University of Medical Science , Tehran, Iran

**Keywords:** newborns, neonatal intensive care unit, counseling

## Abstract

**Background**: Pressures and tensions in everyone’s life can cause a wide range of mental disorders such as anxiety. One of these tensions is the birth of a baby who requires special care, which can cause personal and social problems for the mother if no appropriate measures are taken to help them. The present study was conducted to determine the effect of cognitive-behavioral counseling on anxiety in the mothers of infants in the Neonatal Intensive Care Unit.

**Methods**: This randomized controlled trial recruited 90 women presenting to Kowsar Hospital in Qazvin in 2016. They were enrolled by convenience sampling and randomly assigned to control and intervention groups. Eight sessions were held for each group. Beck Anxiety Inventory was filled by mothers at the beginning of intervention, at the end of the eighth session and three weeks after the intervention. The data was analyzed by generalized estimating equations (GEE) method.

**Results**: According to the results, maternal anxiety showed no significant differences between the two groups before intervention (p = 0.408 and p = 0.881). Based on GEE test, the mean score of anxiety was significantly different in the two groups (p = 0.026) immediately and three weeks after the intervention in that it was lower in the intervention group. Friedman test results also confirmed the reducing trend of mean score of anxiety in the three stages (p = 0.000).

**Conclusions**: Counseling has a positive effect on reducing the anxiety of mothers of children with special needs, therefore it can be used to improve their condition.

## Introduction

 Anxiety is one of the most common adjustment disorders in all societies, which has a global prevalence of 14% and a prevalence of 20.8% in Iran ^1, 2^.

Women’s life is made up of stages and events such as menstruation, pregnancy and childbirth and their associated psychological and physical changes, which make women more vulnerable than men in the face of the stresses of life ^3, 4^. One of the most important and common causes of stress and anxiety in women during the reproductive years is concern about the health of their fetus and future child ^4^.

The birth of an infant who requires intensive care is considered a serious crisis in the mother’s life. Uncertainty about survival, future life and consequences, duration of hospitalization and taking care of the child at home are some of the mother's concerns that may lead to confusion, frustration, fear and ultimately anxiety ^5^. If these parents receive no support in coping with these circumstances, they may adopt ineffective coping strategies ^6^, which can affect the quality of the mother’s personal and social life and her ability to take care of the infant and may thus increase her anxiety ^6– 8^. Under such circumstances, the mother needs emotional support from her spouse and even the society, and providing this support can diminish her anxiety ^9^. Paying attention to the mother's needs and identifying the factors causing stress in her are necessary to reduce her anxiety ^10^.

The strategies for coping with and treating this anxiety include pharmacological and non-pharmacological therapies. A wide variety of non-pharmacological interventions are currently available that can be more effective than mere pharmacological therapy; for instance, counseling and anxiety management training ^11^.

Counseling refers to the professional relationship established between a trained counselor and a client. This relationship is built to help the client better grasp her own views of her living environment and to teach her how to achieve her personal goals by choosing tested and meaningful personal strategies and also solve her emotional and interpersonal problems ^12^, which can be performed individually or in a group setting. Group counseling is more effective than individual counseling in critical conditions when mental pressure is accompanied by severe disappointment and the individual finds herself feeling broken, inadequate, helpless and feels fear and failure ^12^. Several studies have investigated the effectiveness of counseling with a cognitive-behavioral approach; for instance, Seyed Gholami *et al.* found that this method is effective in reducing anxiety sensitivity in divorced women ^13^.

Cognitive-behavioral therapy is a combination of cognitive and behavioral approaches to help the patient identify and correct her distorted patterns of thinking and inefficient behaviors and is carried out with regular discussions and carefully-organized behavioral assignments ^14^. In other words, the patient is encouraged in this method to consider the relationship between her negative thoughts and feelings as assumptions that ought to be put to test and to use behaviors resulting from negative thoughts as a benchmark for assessing the validity or accuracy of these thoughts ^15^.

Since midwives are a group of healthcare workers that have an important role in providing counseling and health training not just to women but to entire families and the community, they can play a significant part in the provision of these interventions ^16^. Given the importance of addressing the health of mothers of children requiring special care, the present study was conducted to determine the effect of cognitive-behavioral counseling on anxiety in the mothers of infants in the Neonatal Intensive Care Unit (NICU).

## Materials and methods

### Design

The present parallel randomized controlled trial was approved by the Ethics Committee of Alborz University of Medical Sciences with the code abzums.rec.1395.13, dated May 11 ^th^ 2016, Irct ID: IRCT2016051427728N1 (registered on June 28 ^th^ 2016). It was conducted on 90 eligible women with infants hospitalized at the NICU of Kowsar Hospital in Qazvin in August – October 2016.

### Sample

The sample size was estimated as 42 per group based on the existing literature ^2, 4, 6^ and the pre- and post-intervention standard deviations of 3.65 and 2.71 in order to reach a mean difference (reduction in anxiety score) of 1.96 with the following specifications and using the sample size equation (N=(z1−α2⁄−z1−β) ^2^( *SD*1+ *SD*2) ^2^/ *d*2) for comparing two means; the estimated sample size was increased to 45 per group to take account of potential attrition 10%.

The study inclusion criteria were: 1)Being an Iranian woman, 2) having at least a high school diploma, 3) having had a preterm birth (less than 37 weeks) or low-birth-weight infant (weighing less than 2500 grams) or both with a minimum NICU stay of one month, 4) suffering from anxiety based on Beck's Anxiety Inventory ^17^, 5) no self-reported psychiatric disorders, 6) no use of anxiolytic medications, 7) willingness to participate in the study, 8) no history of adverse life events such as the death of a first-degree relative in the past six months and 9) no history of giving birth to a child with special diseases or severe abnormalities. ( Supplementary file 1, Supplementary file 2).

### Data and measures

The tools used in this study included a personal-demographic checklist as well as Beck's Anxiety Inventory (BAI), which contains 21 items scored based on a 4-point Likert scale (with responses including ‘not at all’, ‘mildly’, ‘moderately’ and ‘severely’). BAI is an international scale with a confirmed validity and reliability according to several studies ^18^. This questionnaire was completed by the participants of this study before and immediately after the end of the counseling sessions and also three weeks after the intervention.

### Procedures

The study began after obtaining the necessary permissions from the Research Council, Ethics Committee and Director of Alborz University of Medical Sciences as well as the director and department head of Kowsar Hospital in and the registration of the project at the Iranian Registry of Clinical Trials (IRCT). For sampling, the researcher (MK) visited the NICU of Kowsar Hospital and selected all the eligible mothers through convenience sampling and briefed them on the study objectives and obtained their written consent for entering the study if they were willing to participate. Permuted block randomization was used to randomly allocate participants into the trial group while maintaining a balance across the groups. Each block had a specified number of randomly-ordered trial assignments, so the mothers were divided into a trial group (anxiety counseling + routine care counseling) and a control group (routine care counseling). Both groups received eight sessions of routine counseling (routine neonatal care), twice per week for four weeks. The subjects discussed in the routine care counseling sessions included:

**Session 1**: Information about the hospitalized infant, such as the type of disease and the diagnostic and therapy methods used. **Session 2**: Information about the disease symptoms and consequences. **Session 3**: Obtaining knowledge and skills about nutrition. **Session 4**: Obtaining knowledge and skills about moving and positioning. **Session 5**: Obtaining knowledge and skills about hygiene and infection control. **Session 6**: Obtaining knowledge and skills about temperature and how to clothe the infant. **Session 7**: Obtaining knowledge and skills about the infant’s behavior. **Session 8**: Obtaining knowledge and skills on how to interact with the infant.

In addition to the routine counseling, the trial group also received anxiety counseling with a cognitive-behavioral approach. It should be noted that the researcher (MK) had received prior training on CBT counseling and participants’ appropriate mental state and their diagnosis of anxiety were confirmed under the supervision of a clinical psychologist. After routine counseling for 50 minutes, the trial group also received anxiety counseling with the following subjects: **Session 1**: Establishing relationships among the mothers, learning the rules of the group, determining the goals of group therapy and getting feedback. **Session 2**: The psychological recounting of feelings and thoughts about the birth of their hospitalized infant in the context of a support group, emotional adjustment and release in a supportive environment and help homogenize the group members’ feelings. **Session 3**: Reviewing the signs of stress and introducing the concept of stress relief for obviating signs of stirred emotions. **Session 4**: Assessing the effect of cognition and thoughts on stress responses and helping mothers recognize their negative inner self-talk and introducing the importance of coping skills for stress management and assessing how people cope with problems. **Session 5**: Reviewing stressful self-talk and encouraging the mothers to turn self-talk into effective coping and going over previous stress relief exercises. **Session 6**: Problem-solving training and extracting a description of the problem from every member of the group. **Session 7**: Providing alternative solutions, assessing the solutions and using the best one. **Session 8**: Assessing the efficacy of the solutions used and their readjustment if necessary.

BAI was completed by the mothers at the beginning of the first session, the end of the eighth session and three weeks after the intervention (for the final follow-up).

The data collected were analyzed in SPSS-19 using the Friedman test and the General Estimating Equations (GEE). Statistical hypotheses were verified at the level of p<0.05.

## Results

In the course of the study, nine mothers were excluded from the study, including three from the trial group (one due to her infant’s death and two for failing to complete the sessions) and six from the control group (for failing to complete the sessions). The study thus ended with 81 participants ( Supplementary file 1). Dataset 1

Raw data obtained from Beck’s Anxiety Inventory (BAI) Group: 1=case 2=controlJob: 1= Housekeeper, 2= teacher, 3=Engineer, 5= Employee, 4= otherEducation: 1,2= Associate Degree, 3= BS, 4= MS/PhDanxA1= Anxiety quetion1 before interventionanxB1= Anxiety quetion1 Immediately after interventionanxC1= Anxiety quetion1 Three weeks after intervention
Copyright: © 2017 Koochaki M et al. 2017 Data associated with the article are available under the terms of the Creative Commons Zero "No rights reserved" data waiver (CC0 1.0 Public domain dedication).

The results obtained showed no significant differences between the trial and control groups in terms of age, education, occupation and income and the groups were thus matching in terms of these variables ( Table 1). Moreover, the groups were not significantly different in terms of the mean score of anxiety before the intervention (p=0.408) and had the same level of anxiety before the interventions begun.

**Table 1.** The distribution of the demographic details of the mothers of infants.

Variables studied		Studied groups		p-value
		control group	Intervention group	
		F(%)	F(%)	
Level of Education	Associate Degree	25(64.1)	23(54.7)	
	BS	10(25.6)	18(42.9)	
	MS/PhD	4(10.3)	1(2.4)	
Mean rank **		39.46	42.43	0.528
Job Status	Housekeeper	25(64.1)	25(59.5)	
	teacher	1 (2.6)	3 (7.1)	
	Engineer	2 (5.1)	1 (2.4)	
	Employee	6 (15.4)	7 (16.7)	
	other	5 (12.8)	5 (12.8)	
Mean rank **		40.18	41.76	0.728
income	0-1000000R	27(69.2)	26(61.9)	
	1000000- 1500000R	9(23.1)	12(28.6)	
	≥1500000R	3(7.7)	4(5.9)	
Mean rank **		40.38	41.57	0.731
		mean±SD *		
age		22.87±3.799	23.83±3.831	0.210
Anxiety scores		20.67 ± 6.791	19.45 ± 6.345	0.408

p-value <0.05 *:Independent t-test **:Mann–Whitney *U* test

A comparison of the mean score of anxiety between the two groups over three measurement phase using the GEE showed a significant difference between them in the trend of changes in anxiety ( Table 2). The results showed that the score of anxiety reduced immediately after the intervention compared to before in both groups, and although this score had increased in the three-week follow-up compared to immediately after the intervention, it was still lower than the scores obtained before the intervention.

**Table 2.** A comparison of the level of anxiety in the mothers of infants before, immediately after and three weeks after the intervention in two groups.

groups	Mean ± SD			GEE test
	Before intervention	Immediately after the intervention	Three weeks after intervention	
Intervention group	19.45 ± 6.345	9.7 ± 3.645	11.52 ± 4.528	P=0.026
control group	20.67 ± 6.791	8.95 ± 3.720	15.46 ± 5.062	

p-value <0.05

The trend of changes in the score of anxiety in the trial and control groups was analyzed using the Friedman test, which revealed a significant difference over the three measurement occasions and thus suggests the effectiveness of the intervention in the trial group ( Table 3).

**Table 3.** A comparison of the level of anxiety in the mothers of infants hospitalized before, immediately after and three weeks in each groups.

groups	Mean ± SD			Friedman test
	Before intervention	Immediately after the intervention	Three weeks after intervention	
Intervention group	19.45 ± 6.345	9.7 ± 3.645	11.52 ± 4.528	P<0.001
control group	20.67 ± 6.791	8.95 ± 3.720	15.46 ± 5.062	P<0.001

p-value <0.05

## Discussion

Anxiety is a highly unpleasant, generalized and often significant feeling of concern that is accompanied by one or several collective feelings and common symptoms, such as feeling discharged, shortness of breath, heart palpitations, headache, perspiration, sudden urinary incontinence and restlessness ^19^. The present findings showed that cognitive-behavioral counseling reduces anxiety in mothers of children in a NICU. No significant differences were observed between the two groups in the mean score of anxiety before the intervention. The GEE showed a significant difference between the two groups in the mean score of anxiety immediately and three weeks after the intervention, as this mean score was lower in the trial group. The Friedman test showed significant differences in the mean score of anxiety in each group before, immediately and three weeks after the intervention. Comparing the mean scores showed a reduction in anxiety. The results of these two tests showed a reduction in the mean level of anxiety in both the trial and control groups; however, the reduction was higher in the trial group. As a result, both routine and cognitive-behavioral counseling can help reduce anxiety levels, but the reduction achieved is greater and more long-lasting with the latter.

The present findings are consistent with the results of other studies, Dehshiri found cognitive-behavioral interventions to be significantly effective in reducing anxiety and concern in patients with generalized anxiety disorder ^14^. Patients with generalized anxiety disorder lack the ability to regulate their basic emotions, and cognitive-behavioral counseling teaches them techniques to help control the physiological components of their anxiety and modify their inaccurate interpretations of the events around them and thus helps reduce their anxiety; these techniques include diaphragmatic breathing and progressive/mental relaxation ^14^. A study by Danae *et al.* confirmed the effectiveness of this intervention in reducing anxiety. According to their study, cognitive-behavioral counseling reduces anxiety in patients with migraine headaches by correcting their self-induced negative thoughts (concern about other’s misunderstandings, etc.) and fundamental beliefs and by teaching problem-solving skills ^18^.

Other studies have also shown the effectiveness of cognitive-behavioral counseling in reducing anxiety by identifying inefficient thoughts and correcting them and by teaching diaphragmatic or deep breathing and progressive/mental relaxation techniques as well as problem solving skills ^20, 21^.

Cognitive-behavioral therapy is based on the assumption that, instead of constructive behavior and appropriate coping responses, anxious people are rather predisposed to the perception of threat and avoidance coping responses, which lead to the persistence of anxiety and concern ^22^. The daily observation of the behaviors of people who react differently to the same situation and the formation of judgments, evaluations, expectations, perceptions and other mental processes concerned with the individual's awareness can help change the individual’s own behavior and treatment in the face of different situations. Cognitive-behavioral therapy helps patients take the most adaptive and reasonable interpretation and adopt behaviors that match their new perspective ^23^.

The trial group received training on how to be aware of the physiological and emotional signs of anxiety in themselves and learned more about their own behavioral patterns in response to anxiety and about how to perform a relaxation technique with the first signs of anxiety so as to reduce anxiety. The control of self-induced thoughts may be another reason for the reduced anxiety in the trial group. Learning meditative relaxation and cognitive techniques helps the individual deal effectively with her anxiety ^24^.

As noted earlier, midwives are a group of healthcare workers that have an important role in providing counseling and health training not just to women but to entire families and the community, and they can therefore play a significant part in the provision of these interventions ^19^. Facilitating the provision of this type of counseling to mothers can therefore help reduce their anxiety and improve their quality of life and infant care skills.

### Limitations

 As for the limitations, the participants with their conditions (i.e. have a infants in the NICU), may have affected their response to the questions; however, this point was beyond the researcher's control, even though attempts were made to obviate this limitation by imposing certain inclusion and exclusion criteria and ensuring homogeneity between the two groups.

## Conclusion

The present findings showed that both routine and cognitive-behavioral counseling can reduce anxiety in mothers with infants in the NICU; however, this reduction was greater and more long-lasting in the cognitive-behavioral counseling group. Given the role of medical personnel, especially midwives, in communicating with mothers and helping them deal with their problems, efforts should be made to create a conducive environment for counseling in order to help reduce anxiety and stress in mothers with special conditions and ultimately improve their quality of life and readiness for taking care of their infants.

## Data availability

The data referenced by this article are under copyright with the following copyright statement: Copyright: © 2017 Koochaki M et al.

Data associated with the article are available under the terms of the Creative Commons Zero "No rights reserved" data waiver (CC0 1.0 Public domain dedication). 

Dataset 1: Raw data obtained from Beck's Anxiety Inventory (BAI)

- Group: 1=case 2=control
- Job: 1= Housekeeper, 2= teacher, 3= Engineer, 5= Employee, 4= other
- Education: 1,2= Associate Degree, 3= BS, 4= MS/PhD
- anxA1=Anxiety quetion1 before intervention
- anxB1= Anxiety quetion1 Immediately after intervention
- anxC1= Anxiety quetion1 Three weeks after intervention

10.5256/f1000research.12539.d176433 ^25^

## Ethics and consent

This study was approved by the Ethics Committee of Alborz University of Medical Sciences with the code abzums.rec.1395.13, dated May 11 ^th^ 2016, Irct ID: IRCT2016051427728N1 (registration date June 28 ^th^ 2016). All participants provided written informed consent to participate in the study.

## Author details

M. Koochaki is an MSc student in Consultation on Midwifery, at Alborz University of Medical Sciences, Karaj, Iran.

Z. Mahmoodi is an Assistant Professor of Non-Communicable Disease Research Center, at Alborz University of Medical Sciences, Karaj, Iran.

S. Esmaelzadeh – Saeieh is an Assistant Professor of Nursing & Midwifery, and a Faculty member of Alborz University of Medical Sciences, Karaj, Iran.

K. Kabir is an Assistant Professor of Community Medicine, at the School of Medicine; and a Faculty member of Alborz University of Medical Sciences, Karaj, Iran.

M. Tehranizadeh is an Assistant Professor and Faculty member of Payame Noor University, Kara, Iran.

M. Dolatian is an Assistant Professor at the Department of Midwifery and Reproductive Health, and a Faculty member of Shahid Beheshti University of Medical Science, Tehran, Iran.

## Supplementary material

Supplementary file 1

Completed CONSORT flow diagram.

Supplementary file2:

Completed CONSORT check list.
