# Supplementary material for: The effect of cognitive-behavioral counseling on anxiety in the mothers of infants in the NICU: A randomized controlled trial
Source: F1000Res. 2017 Sep 12;6:1679. [Version 1] doi: 10.12688/f1000research.12539.1 (PMC5605950; doi:10.12688/f1000research.12539.1)
Supplement: Supplementary file 2 [file f1000research-6-13577-s0001.tgz › 466ead59-bcaf-452e-9387-c51f2bb4fb51.docx]

Analysis N=39

Analysis N=42

Control group N=39

Intervention group N=42

Follow up

Out of the study (n=6)

Non-complication of the class

Out of the study (n=3)

Infant death (n=1)

Non-complication of the class (n=2)

Routine care (N=45)

Routine care+ Cognitive-behavioral counseling (N=45)

Samples Available (N=90)

Assessments of eligible (N=90)

Enrollment

Random allocation

N=90

n

Figure1: consort Flow Diagram
