# Supplementary material for: The effect of cognitive-behavioral counseling on anxiety in the mothers of infants in the NICU:  A randomized controlled trial
Source: F1000Res. 2017 Sep 12;6:1679. [Version 1] doi: 10.12688/f1000research.12539.1 (PMC5605950; doi:10.12688/f1000research.12539.1)
Supplement: Peer review history

# Peer review: Referee response for version 1

**DOI:** 10.5256/f1000research.13577.r25999

**Published:** 2017-09-27

**Authors:** Razieh Bagherzadeh

**Affiliations:** 1 Department of Midwifery, School of Nursing and Midwifery, Bushehr University of Medical Sciences, Bushehr, Iran

I appreciate the authors for their initiative. They have selected an interesting topic for study. Certain points are given below so that they can work on them to make it better.

1. In result section of abstract (line 2 of result) authors have reported two P value for non-differences in maternal anxiety between the two groups before intervention. Why two P value?
2. Result section of abstract (line 4 and 5 of result) does not match with results in Table 2. Please see mean of anxiety score immediately after the intervention in two groups.
3. In sample size calculation, how much α and β is considered? Please mentioned.
4. Please correct attrition rate (10%.??)
5. In Table 1, income per week or month??
6. In table 1: why mean rank for Job Status? Mean rank for nominal variable is not correct.
7. Results (In Table 2 and 3) show reduction of anxiety score in both groups and further reduction in the control group Immediately after the intervention, but in discussion section (paragraph 1) authors have written: "The GEE showed a significant difference between the two groups in the mean score of anxiety immediately and three weeks after the intervention, as this mean score was lower in the trial group." Reported results did not indicate whether the statistically significant mean difference between the two groups is Immediately after the intervention  and/or in lasting? Discussion section will be according to results.

I have read this submission. I believe that I have an appropriate level of expertise to confirm that it is of an acceptable scientific standard.

# Peer review: Referee response for version 1

**DOI:** 10.5256/f1000research.13577.r25941

**Published:** 2017-09-15

**Authors:** Katayoun Falahat

**Affiliations:** 1 Social Determinants of Health Research Center, University of Social welfare and Rehabilitation, Tehran, Iran; 2 Ministry of Health and Medical Education, Tehran, Iran

ّFirst of all, Thank you so much to choose me as a reviewer.

 The research topic is new and very interesting. However, reading this article causes some question

1. Would you please explain more about GEE? Why do you use this analysis method? I think the description of this method is empty in Data analysis Section.
2. Has the researcher have a clinical experience to perform CBT in different subjects? How long did each session of CBT take? And how did the CBT session perform? Individually of in a group?

Thank you so much again.

I have read this submission. I believe that I have an appropriate level of expertise to confirm that it is of an acceptable scientific standard.

# Response

**Published:** 2017-09-15

**Authors:** Zohreh Mahmoodi

**Affiliations:** Assistant Professor, Non-communicable Disease Research Center ,Alborz University of Medical Sciences, Karaj, Iran, Iran

Dear Editor Thank you for your decision.I answer the question:

1. Would you please explain more about GEE? Why do you use this analysis method? I think the description of this method is empty in Data analysis Section.

Answer: GEE is a Generalized Estimating Equations and the Description data is in Table 1

1. Has the researcher have a clinical experience to perform CBT in different subjects? How long did each session of CBT take? And how did the CBT session perform? Individually of in a group?

Answer: Yes the researcher had received prior training on CBT counseling.

Both groups received eight sessions of routine counseling (routine neonatal care), twice per week for four weeks and each session was 50 minutes

Regards.

# Peer review: Referee response for version 1

**DOI:** 10.5256/f1000research.13577.r25942

**Published:** 2017-09-15

**Authors:** Soheila Pirdadeh Beiranvand Beiranvand

**Affiliations:** 1 Reproductive Health, Department of Midwifery, Faculty of Nursing and Midwifery, Lorestan University of Medical Sciences, Khorramabad, Iran

This is a well-conducted and written study. I have the following comments:

  

1. Please explain main reasons why you used Cognitive-behavioral therapy for this subject?
2. In my opinion, it is better to write ethical code in the end of method not in design.
3. Please explain about validity and reliability of questionnaire in paper

I have read this submission. I believe that I have an appropriate level of expertise to confirm that it is of an acceptable scientific standard.

# Response

**Published:** 2017-09-15

**Authors:** Zohreh Mahmoodi

**Affiliations:** Assistant Professor, Non-communicable Disease Research Center ,Alborz University of Medical Sciences, Karaj, Iran, Iran

Dear Editor ,

Thank you for your decision. I answered your questions:

- Please explain main reasons why you used Cognitive-behavioral therapy for this subject?

Answer: Cognitive-behavioral therapy is a combination of cognitive and behavioral approaches to help the patient identify and correct her distorted patterns of thinking and inefficient behaviors and is carried out with regular discussions and carefully-organized behavioral assignments

- In my opinion, it is better to write ethical code in the end of method not in design.

Answer: We did it as the editor Suggested.

- Please explain about validity and reliability of questionnaire in paper

BAI is an international scale with a confirmed validity and reliability according to several studies and we got a reference in Data and measures
